# THAP7-AS1 sponges miR-4286 to upregulate PFKFB2, accelerating aerobic glycolysis and aggravating liver cancer progression

**DOI:** 10.3389/fcell.2026.1903996

**Published:** 2026-08-10

**Authors:** Qiongqing Huang, Jingjing Xia, Zhengui Liang, LiNing Huang, Huanjie Zhou, Liping Dou, Chao Ou

**Affiliations:** 1 Department of Clinical Laboratory, Guangxi Medical University Cancer Hospital, Nanning, Guangxi, China; 2 Department of Medical Science Laboratory, The Fourth Affiliated Hospital of Guangxi Medical University, Liuzhou, Guangxi, China

**Keywords:** aerobic glycolysis, liver cancer, miR-4286, PFKFB2, THAP7-AS1

## Abstract

**Background:**

Liver cancer ranks among the most prevalent and deadliest malignancies worldwide. As one of the largest metabolic organs, the liver undergoes profound metabolic reprogramming, encompassing oxidative phosphorylation and aerobic glycolysis (the Warburg effect)-a hallmark of liver cancer progression that drives tumor growth, metastasis, and chemoresistance. Accumulating evidence indicates that long non-coding RNAs (lncRNAs) play critical roles in cancer biology. In this study, we identify THAP7-AS1 as an oncogenic lncRNA that regulates aerobic glycolysis and promotes liver cancer tumorigenesis.

**Methods:**

RNA sequencing was employed to screen for differentially expressed genes in the serum of liver cancer patients versus healthy controls. The functional roles of THAP7-AS1 in aerobic glycolysis and liver cancer progression were investigated using CCK-8, colony formation, EDU incorporation, flow cytometry, and xenograft tumor assays. The underlying molecular mechanisms were explored via fluorescence *in situ* hybridization (FISH), RNA immunoprecipitation (RIP), and dual-luciferase reporter assays.

**Results:**

High THAP7-AS1 expression was significantly correlated with poor prognosis in liver cancer patients. Functionally, both *in vitro* and *in vivo* experiments confirmed that THAP7-AS1 promotes liver cancer cell growth. Moreover, THAP7-AS1 substantially enhanced glycolytic activity in liver cancer cells. Mechanistically, THAP7-AS1 functions as a competitive endogenous RNA (ceRNA) by sponging miR-4286, which in turn negatively regulates PFKFB2 and plays a pivotal role in aerobic glycolysis.

**Conclusion:**

THAP7-AS1 modulates aerobic glycolysis in liver cancer cells through the miR-4286/PFKFB2 axis. Collectively, our findings suggest that THAP7-AS1 holds promise as a serum diagnostic biomarker and a potential therapeutic target for overcoming liver cancer progression.

## Introduction

Liver cancer is a highly malignant and common fatal malignant tumor worldwide (Bray et al., 2018). Despite improvements in liver cancer prognosis achieved by surgical resection and sorafenib-based therapies, the tumor’s insidious onset and early metastatic potential lead to postoperative recurrence and metastasis in over 70% of patients, even among those diagnosed at an early stage (Marrero et al., 2018). Thus, there is an urgent need to elucidate the mechanisms underlying liver cancer initiation and progression, to identify reliable diagnostic biomarkers and more effective therapeutic strategies, and ultimately to prevent recurrence and improve patient survival.

In the 1920s, Otto Warburg first demonstrated that, unlike normal cells that rely on oxidative phosphorylation, most tumor cells preferentially convert glucose to lactate even under aerobic conditions. This metabolic reprogramming, termed the “Warburg effect,” is also known as aerobic glycolysis (Koppenol et al., 2011; DeBerardinis et al., 2008; Hanahan and Weinberg, 2011; Hsu and Sabatini, 2008). The intermediates generated during aerobic glycolysis serve as precursors for the biosynthesis of macromolecules in tumor cells. Moreover, the enhanced glucose uptake and extensive lactate production provide carbon sources that fuel anabolic processes essential for cell proliferation, thereby supporting rapid growth, invasion, metastasis, and adaptation to the tumor microenvironment (Bhattacharya et al., 2016). Indeed, as one of the largest metabolic organs, the liver also functions as the primary site of glucose metabolism. Extensive studies have reported that aerobic glycolysis exerts multifaceted regulatory effects in liver cancer, promoting tumor cell survival and proliferation, facilitating invasion and metastasis, driving angiogenesis, and contributing to both immune evasion and chemoresistance (Huang et al., 2024; Balihodzic et al., 2021).

LncRNAs are defined as non-coding RNA molecules longer than 200 nucleotides (nt) that possess little to no protein-encoding capacity (Derrien et al., 2012). However, aberrant lncRNA expression is critically involved in various physiological and pathological processes, ranging from development, immune response, and metabolism to tumor initiation and progression (Statello et al., 2021; Rao et al., 2017). Emerging evidence has revealed that lncRNAs can function as competitive endogenous RNAs (ceRNAs) by interacting with miRNA response elements (MREs), thereby modulating aerobic glycolysis and playing a critical role in tumor initiation and progression. For example, LncRNA KCNQ1OT1 sponges miR-34c-5p to promote osteosarcoma growth through enhanced aerobic glycolysis by ALDOA (Shen et al., 2020). LINC01123, a c-Myc-activated long non-coding RNA, promotes the proliferation and aerobic glycolysis of non-small cell lung cancer through the miR-199a-5p/c-Myc axis (Hua et al., 2019). Collectively, these findings offer a compelling rationale for our hypothesis that lncRNAs may regulate glucose metabolism in cancer cells via the ceRNA mechanism, thereby promoting liver cancer progression.

In recent years, gene chip and transcriptome sequencing technologies have been widely applied in biomedical research owing to their high throughput and efficiency, facilitating the screening of key genes and elucidation of molecular mechanisms across various tumor models. Among these tools, the ceRNA microarray enables simultaneous screening and analysis of differentially expressed genes including mRNAs, lncRNAs, and circRNAs between experimental and control groups, thereby yielding expression data at all three levels (Li et al., 2022; Plitzko and Loesgen, 2018). Using ceRNA microarray and miRNA sequencing, we identified candidate ceRNAs for further investigation. In the initial phase of this study, serum samples from liver cancer patients and healthy controls were subjected to ceRNA microarray and miRNA sequencing, followed by construction of a ceRNA network comprising differentially expressed lncRNAs, miRNAs, and mRNAs. Subsequently, to screen for candidate lncRNAs and miRNAs involved in the regulation of glycolysis, we first analyzed differentially expressed lncRNAs, miRNAs, and mRNAs based on sequencing data. Intersection analysis was performed between the mRNAs within this regulatory network and the “glycolysis” gene set obtained from GSEA. Concurrently, bioinformatic prediction using TargetScan and miRanda revealed that THAP7-AS1 harbors an endogenous MRE complementary to miR-4286, enabling direct binding to miR-4286, and that miR-4286 binds with high specificity to the 3′-UTR region of PFKFB2 (6-Phosphofructo-2-Kinase/Fructose-2,6-Biphosphatase 2), a core rate-limiting enzyme in glycolysis. Collectively, the THAP7-AS1/miR-4286/PFKFB2 axis was identified as a potential ceRNA mechanism that promotes liver cancer progression via regulation of aerobic glycolysis. These findings prompted us to perform further functional experiments.

To date, reports on THAP7-AS1 remain relatively limited. The only available study has shown that THAP7-AS1 functions as an oncogenic factor in gastric cancer, where it is stabilized post-transcriptionally via METTL3-mediated m6A modification, thereby promoting CUL4B nuclear translocation and exerting its pro-tumorigenic effects (Liu et al., 2022). THAP7-AS1 orchestrates IGF2BP3-m6A-dependent CCN2 mRNA stabilization to promote lymphatic metastasis in bladder cancer (Li et al., 2026). However, the precise role and molecular mechanism of THAP7-AS1 in liver cancer remain largely unexplored.

In this study, we demonstrate that THAP7-AS1 is upregulated in liver cancer and promotes tumor growth by enhancing aerobic glycolysis. Mechanistically, THAP7-AS1 competes with PFKFB2, a key glycolytic enzyme-encoding mRNA, for binding to miR-4286, thereby relieving the miR-4286-mediated suppression of PFKFB2 and leading to increased PFKFB2 expression and glycolytic activity. Collectively, the THAP7-AS1/miR-4286/PFKFB2/MAPK/ERK1/2 axis may represent a novel therapeutic target for liver cancer.

## Materials and methods

### Patients and specimens

Cohort 1: From November 2021 to January 2022, serum samples were collected from liver cancer patients (n = 8) and healthy controls (n = 4) prior to surgery at the Department of Laboratory Medicine, Affiliated Cancer Hospital of Guangxi Medical University. Venous blood samples were collected in dry tubes and centrifuged at 3,000 × g for 5 min to separate the serum. Each serum sample was aliquoted and stored at −80 °C for subsequent analysis.

Cohort 2: Liver cancer tissues (n = 40) and adjacent non-tumor tissues (n = 40) were obtained from surgical specimens archived at the Affiliated Cancer Hospital of Guangxi Medical University between January 2014 and December 2018. Inclusion criteria included histopathologically confirmed liver cancer and availability of complete clinical and pathological data. Patients with other malignancies or those who had received preoperative chemotherapy or radiotherapy were excluded. Tissues were stored at −80 °C until use. Clinical follow-up data were collected, and overall survival (OS) was defined as the time interval from the date of surgery to the date of death or the last follow-up. For Kaplan-Meier survival analysis, a total of 60 liver cancer patients with complete clinical follow-up data from our medical center were included. Patients were separated into high and low THAP7-AS1 groups using the median serum expression level of THAP7-AS1 as the cutoff value. Log-rank test was used to compare overall survival differences between two groups, and hazard ratio (HR) with 95% confidence interval was calculated. This study was approved by the Ethics Committee of the Affiliated Cancer Hospital of Guangxi Medical University (Ethics Committee Protocol Number: KY2024472), and written informed consent was obtained from all participants.

### RNA sequencing

Total RNA was extracted from serum samples, followed by RNA sequencing. Raw images were scanned using an Agilent Scanner G5761A (Agilent Technologies), and raw data were processed using Feature Extraction software. Differentially expressed genes (DEGs) were identified using t-test-derived *P*-values, with a threshold of *P* ≤ 0.05 or a fold change≥2. Unsupervised hierarchical clustering was performed on DEGs, and gene expression patterns across samples were visualized using heatmaps.

In addition, transcriptome sequencing was conducted on Huh7 cells transfected with sh-NC or sh-PFKFB2. RNA sequencing was performed using the NovaSeq X Plus platform (Majorbio, Shanghai). Approximately 1 × 10^7^ knockdown and control cells (Huh7-sh-NC and Huh7-sh-PFKFB2) were collected, and total RNA was extracted using TRIzol for library preparation. DEGs were identified based on the criteria log2FC ≥ 1 and adjusted *P* < 0.05. KEGG pathway enrichment analysis was performed to identify potential signaling pathways associated with PFKFB2.

### Cell culture

Human liver cancer cell lines (Huh7, HepG2, SNU-449, PLC), a human hepatocyte cell line (MIHA), and human embryonic kidney cells (HEK293T) were obtained from the Department of Experimental Research, Affiliated Cancer Hospital of Guangxi Medical University. All cell lines were maintained at 37 °C in a humidified atmosphere containing 5% CO2. Cell line authentication was performed via STR profiling, and all lines were confirmed to be mycoplasma-free.

### RNA extraction and reverse transcription-quantitative polymerase chain reaction (RT-qPCR)

Total RNA was extracted from liver cancer tissues and cultured cells using TRIzol reagent (ThermoFisher, USA), followed by reverse transcription into cDNA using a Reverse Transcription cDNA Synthesis Kit (Takara, Japan). To quantify miR-4286 expression, the miRcute miRNA Isolation Kit (Tiangen, China) and miRNA First Strand cDNA Synthesis Kit (Sangon Biotech, China) were used according to the manufacturers’ instructions. qPCR was performed using SYBR Green PCR Master Mix (Takara, Japan), with β-actin and U6 serving as internal controls.

Primer sequences were listed below:

THAP7-AS1 Forward: ACT​CCC​ACA​TCA​CCC​AGT​CCT​ACC Reverse: CCT​ACG​CGC​CCT​GG-TCA​TCT​CA; PFKFB2 Forward: TCA​AAG​TGT​GTG​GAC​AAA​GCC​AGT Reverse: CTC​TTC​A-CAC​ACA​CCA​GGC​ATC; β-actin Forward: TCC​CTG​GAG​AAG​AGC​TAC​GA Reverse: TGAAG-GTAGTTTCGTGGATGC; U6 Forward: CTCGCTTCGGGCAGCACA Reverse: CCT​ACG​C-GCC​CTG​GTC​ATC​TCA.

### Cell transfection

To establish stable THAP7-AS1 and PFKFB2 knockdown cell lines in Huh7 and HepG2 cells, lentiviral vectors were obtained from Han Heng Biology (China). An empty vector was used as a negative control (NC). Cells were transfected and cultured, and 24 h post-transfection, fresh medium was added. Cells were collected 4 days post-transfection and selected with puromycin. Knockdown efficiency was confirmed by RT-qPCR. For overexpression studies, the THAP7-AS1 overexpression vector (THAP7-AS1-OE) was constructed by inserting the THAP7-AS1 sequence into the pDNA3.1(+) vector (GenePharma, China), with an empty vector serving as the negative control (NC-OE). miR-4286 mimics, NC mimics, miR-4286 inhibitor, and NC inhibitor were purchased from Han Heng (China). Transfections were performed using Lipofectamine 3000 (ThermoFisher, USA) at a final concentration of 50nM, and the transfection efficiency was validated by RT-qPCR. ShRNA sequences were listed below:(sh-THAP7-AS1-1)5′-GATCCGG-TCAAGATGCTGAAGATACACTCGAGTGTATCTTCAGCATCTTGACCGTTTTTTTG-3′and (sh-THAP7-AS1-2) 5′GAT​CCG​CCA​GAG​CAA​GAT​AGT​CTA​CTA​ACT​CGA​GTA​GTA​GAC​TAT-CTT​GCT​CTG​GTT​TTT​TG-3′; (sh-PFKFB2) 5′-GAT​CCG​CCA​GAG​CAA​GAT​AGT​CTA​CTA​CT-CGA​GTA​GTA​GAC​TAT​CTT​GCT​CTG​GTT​TTT​TG-3′.

### Cell viability assays

Cell viability was assessed at 0, 24, 48, and 72 h post-transfection using the Cell Counting Kit-8 (US Everbright, China). Cells were seeded at a density of 2,000 cells per well in 100 μL medium in 96-well plates and incubated at 37 °C. After adding 10 μL of CCK-8 solution to each well, cells were incubated for 1 h at 37 °C, and the absorbance at 450 nm was measured.

### Colony formation assays

For colony formation assays, 800 cells were seeded in 6-well plates and cultured for 2 weeks at 37 °C in a humidified atmosphere with 5% CO2. Colonies were washed with PBS, fixed with 4% paraformaldehyde, and stained with 0.1% crystal violet (1 mg/mL) for 30 min. The number of colonies was counted, and the average was calculated from triplicate experiments.

### 5-Ethynyl-2′-deoxyuridine (EdU) incorporation assay

Cell proliferation was evaluated using an EdU kit (RiboBio, China) according to the manufacturer’s instructions. A total of 1 × 10^4^ cells were seeded per well in 96-well plates and incubated at 37 °C with 5% CO2 for 2 h. Cells were fixed with 4% paraformaldehyde and permeabilized with 0.5% Triton X-100. EdU staining was performed using anti-EdU working solution and Hoechst. Fluorescence microscopy was used to capture images, and the EdU incorporation rate was calculated as the ratio of EdU positive cells (blue) to Hoechst positive cells (red).

### Cell apoptosis analysis

Apoptosis was assessed using the Annexin V-APC/7-AAD Apoptosis Detection Kit (MultiSciences, China) and analyzed on a CytoFLEX flow cytometer (Beckman Coulter, USA). Data were processed using FlowJo software.

### Cellular immunofluorescence (IF)

Specific probes targeting THAP7-AS1 and miR-4286 were designed by GenePharma (China). Huh7 cells (1 × 10^4^) were seeded in 48-well plates with glass slides and cultured overnight. Cells were fixed with 4% paraformaldehyde for 15 min at room temperature, followed by overnight hybridization with probe mixtures from the RNA FISH kit. Nuclear staining was performed with DAPI, and confocal microscopy (Zeiss LSM 980, Germany) was used to visualize subcellular localization.

### Glucose uptake and lactate production assays

Extracellular acidification rate (ECAR) and oxygen consumption rate (OCR) were measured using the Seahorse XF24 Extracellular Flux Analyzer (Seahorse Bioscience, Agilent, USA) with the XF Glycolysis Stress Test Kit and XF Cell Mito Stress Test Kit, respectively. Glucose uptake and lactate production were quantified using commercial kits (APPLYGEN, China; Jiancheng, Nanjing, China) according to the manufacturers’ instructions.

### Western blot analysis

Cells were lysed on ice for 30 min using RIPA lysis buffer (Beyotime, China) supplemented with PMSF (Solarbio, China). Lysates were centrifuged at 15,000 × g at 4 °C for 15 min, and the supernatant was boiled at 100 °C for 5 min. Protein concentration was determined using a BCA Protein Assay Kit (Beyotime, China). Proteins were separated by 7.5%–12% SDS-PAGE and transferred to PVDF membranes. Membranes were blocked with 5% skimmed milk for 1 h, incubated with primary antibodies overnight at 4 °C, and then with secondary antibodies for 90 min at room temperature. Protein bands were visualized using an ECL detection kit (4A Biotech, China) and imaged using an analytikjena (Germany) imager.

### RNA binding protein immunoprecipitation (RIP)

RIP was performed using a commercial kit (BersinBio, China) according to the manufacturer’s instructions. Cell lysates were incubated with magnetic beads conjugated with anti-Ago2 (Abmart, China) or anti-IgG (Proteintech, USA). RNA was extracted from immunoprecipitated complexes and analyzed by RT-qPCR and agarose gel electrophoresis to confirm target binding.

### Dual-luciferase reporter assay

Plasmids were constructed by MiaoLing (China). Huh7 and HepG2 cells were co-transfected with miR-4286 mimics or miR-4286 NC and the corresponding luciferase reporter plasmids using Lipofectamine 3000 (Invitrogen, USA). Luciferase activity was measured 40 h post-transfection using the Dual-Luciferase Reporter Assay System (Promega, USA).

### Animal studies

Five-week-old male BALB/c nude mice were obtained from Vital River Laboratory Animal Technology Co., Ltd. (China) and housed under specific pathogen-free conditions. For tumor xenograft experiments, mice were randomly divided into two groups and subcutaneously injected with 1.5 × 10^6^ cells of THAP7-AS1-NC or THAP7-AS1-shRNA (n = 6). Tumor volume was measured every 7 days using the formula: Volume = length × width^2^ × 0.5. On day 35, mice were euthanized, and tumors were collected for weight and volume measurements. Tissues were fixed in 10% formalin and subjected to hematoxylin and eosin (H&E) staining for histological analysis and immunohistochemistry. All animal experiments were approved by the Animal Ethics Committee of Guangxi Medical University.

### Immunohistochemistry (IHC)

Tissues were fixed in 10% formalin, embedded in paraffin, and sectioned. After deparaffinization, rehydration, and antigen retrieval, sections were incubated with primary antibodies. Images were captured using an Axio Imager. Z2 microscope. The immunoreactivity score was calculated by multiplying the staining intensity score by the percentage of positive cells.

### Statistics analysis

Statistical analysis was conducted using GraphPad Prism version 8.0 (GraphPad Software) and SPSS version 22.0 (SPSS, USA). Data are presented as the mean ± standard deviation (SD) from at least three independent experiments. Two-tailed Student’s t-test was used to calculate the statistical significance between groups. One-way ANOVA analysis was performed for more than three groups. Survival analysis was performed using the Kaplan–Meier method and log-rank tests. *P* < 0.05 was considered statistically significant. (*P* > 0.05 = ns, *P* < 0.05 = *, *P* < 0.01 = **, *P* < 0.001 = ***).

## Results

### THAP7-AS1 is upregulated in liver cancer and is associated with a poor prognosis in patients with liver cancer

**FIGURE 1:**
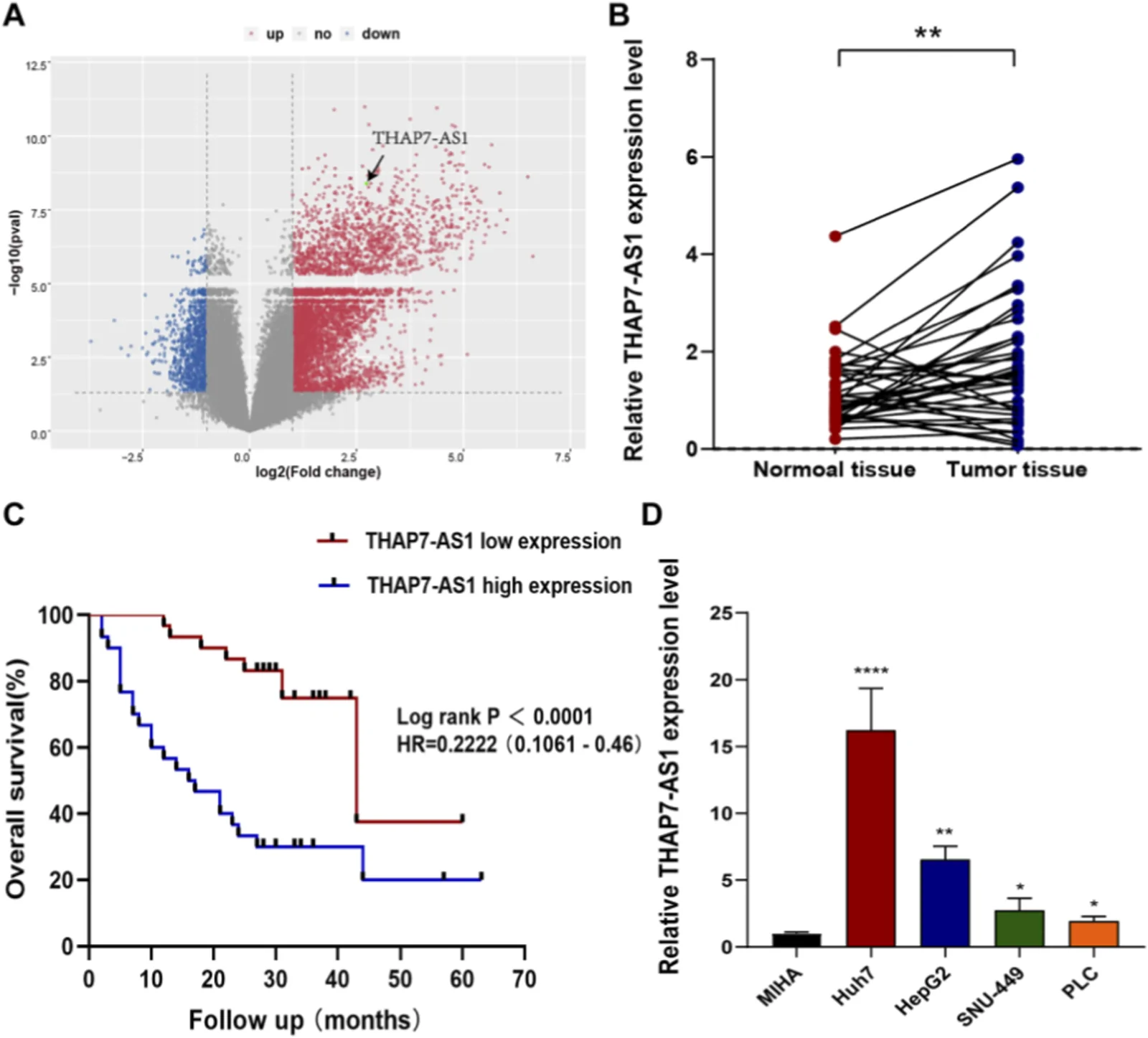
THAP7-AS1 is upregulated in liver cancer and is associated with a poor prognosis in patients with liver cancer. **(A)** Volcano plot showing differentially expressed genes between serum samples from eight liver cancer patients and four normal individuals. The x-axis represents the fold change in read density, and the y-axis represents the adjusted *P*-value. **(B)** Relative expression of THAP7-AS1 in liver cancer tissues and paired adjacent normal tissues detected by qRT-PCR (Student’s t-test). (n = 40, *P* = 0.0012). **(C)** Kaplan-Meier survival analysis revealed that high expression of THAP7-AS1 was correlated with shorter overall survival time in liver cancer patients.(n = 60, *P* < 0.0001, log-rank test). **(D)** Relative THAP7-AS1 RNA expression levels in the normal liver cell line MIHA and various liver cancer lines, as determined by qRT-PCR (One-way ANOVA). *P < 0.05, **P < 0.01, ***P < 0.001, ****P < 0.0001.

Circulating lncRNAs derived from serum have emerged as promising liquid biopsy biomarkers. They offer the advantages of minimally invasive and convenient sample collection, enable dynamic monitoring of therapeutic responses, and facilitate early diagnosis and long-term follow-up assessment (Pardini et al., 2019; You et al., 2023). High-throughput RNA sequencing was performed on serum samples from 8 untreated liver cancer patients and 4 healthy controls. Differentially expressed genes were identified using a t-est, with significance thresholds set at fold change ≥2, *P* ≤ 0.05, and FDR ≤0.05. The expression profile revealed a total of 5,583 differentially expressed genes, including 4,548 upregulated and 1,035 downregulated genes (Figure 1A). Among these, lncRNA THAP7-AS1 drew our attention, as its expression was upregulated in the serum of liver cancer patients. Although its role in gastric cancer progression has been previously reported, its function in liver cancer remains largely unexplored (Liu et al., 2022). Therefore, we aimed to investigate the functional role of THAP7-AS1 in liver cancer. To validate the reliability of the RNA sequencing data, we collected 40 pairs of liver cancer and corresponding adjacent non-tumor tissues and measured THAP7-AS1 expression by RT-qPCR. Consistent with the sequencing results, THAP7-AS1 was significantly upregulated in liver cancer tissues compared with adjacent non-tumor tissues (n = 40, *P* = 0.0012, Figure 1B). For overall survival (OS) analysis, Kaplan-Meier analysis revealed that high THAP7-AS1 expression was significantly associated with worse overall survival (n = 60, *P* < 0.0001, Figure 1C). To further explore its biological function, we first assessed THAP7-AS1 expression in liver cancer cell lines and observed marked upregulation compared with normal MIHA cells (Figure 1D).

These findings suggest that THAP7-AS1 upregulation may contribute to liver cancer progression.

### Knockdown of THAP7-AS1 inhibits the *in vitro* proliferation of liver cancer cells

**FIGURE 2:**
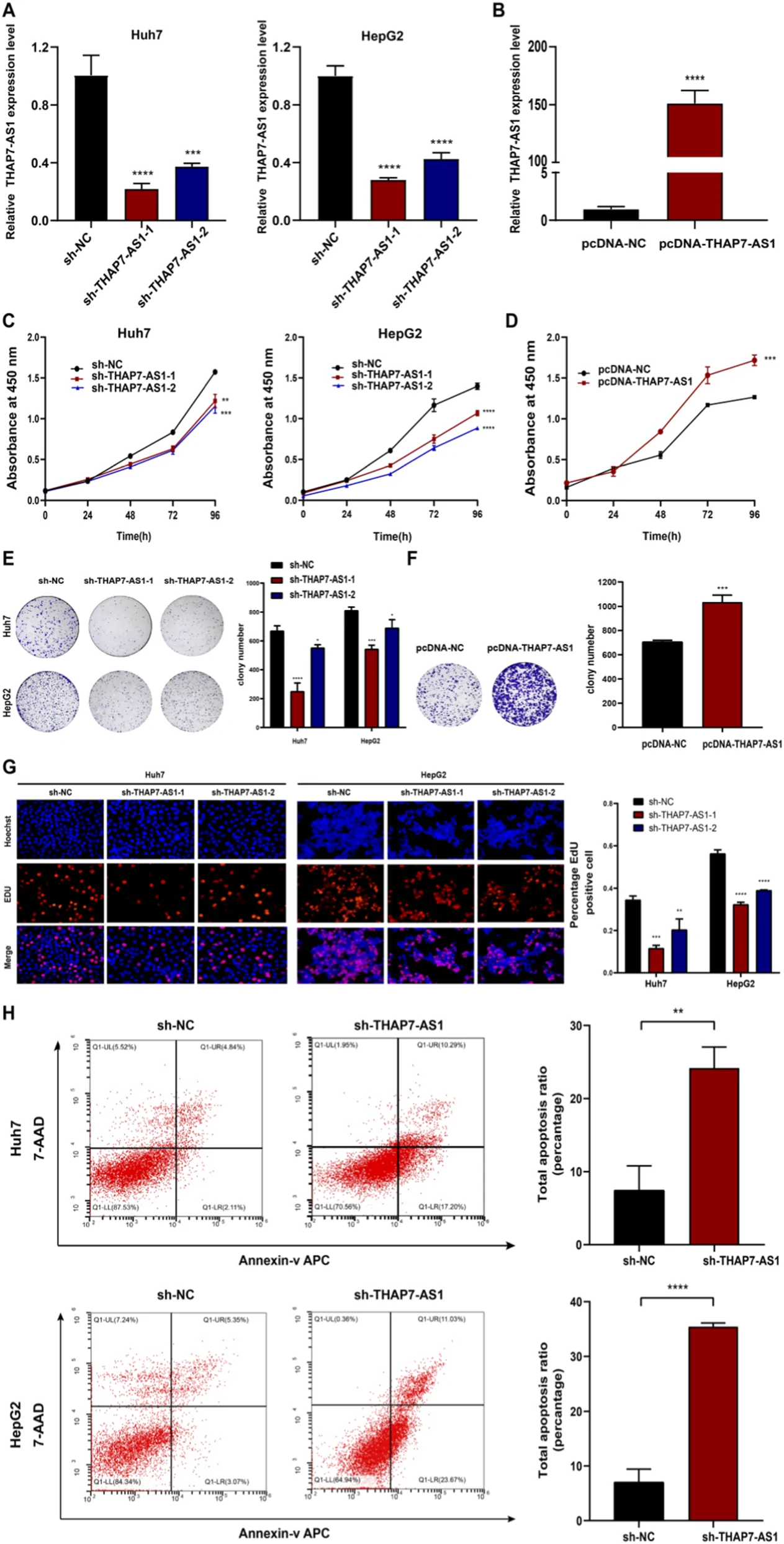
Knockdown of THAP7-AS1 inhibits the *in vitro* proliferation of liver cancer cells. **(A)** THAP7-AS1 expression in control and sh-THAP7-AS1 Huh7 and HepG2 cells, measured by qRT-PCR (One-way ANOVA). **(B)** THAP7-AS1 expression in control and pcDNA-THAP7-AS1 Huh7 cells, measured by qRT-PCR (Student’s t-test). **(C)** The CCK8 method shows that THAP7-AS1 gene knockdown inhibits the proliferation of liver cancer cells (One-way ANOVA). **(D)** The CCK8 method shows that THAP7-AS1 gene overexpression promotes the proliferation of liver cancer cells (Student’s t-test). **(E,F)** Colony formation experiment detects the proliferation ability of liver cancer cells (One-way ANOVA). **(G)** EdU staining and Hoechst nuclear counterstaining in THAP7-AS1-knockdown liver cancer cells, with quantification of EdU-positive cells (One-way ANOVA). **(H)** FCM of apoptosis levels (Student’s t-test).*P < 0.05, **P < 0.01, ***P < 0.001, ****P < 0.0001.

To investigate the biological role of THAP7-AS1 in liver cancer cells, shRNA was employed to effectively silence the gene in Huh7 and HepG2 cell lines. We selected sh-THAP7-AS1-1 and sh-THAP7-AS1-2 for subsequent experiments after the detection of gene knockdown efficacy verified through qRT-PCR (Figure 2A). Conversely, THAP7-AS1 expression was upregulated in Huh7 cells by transfection with a THAP7-AS1 expressing plasmid, and the efficiency of overexpression was confirmed (Figure 2B). Both knockdown and overexpression cell lines were used for subsequent functional assays. CCK-8 and colony formation assays revealed that THAP7-AS1 knockdown significantly suppressed hepatoma cell proliferation, whereas its overexpression promoted proliferation (Figures 2C–F). Consistently, EdU incorporation assays demonstrated that THAP7-AS1 inhibition weakened liver cancer cell proliferation (Figure 2G). Furthermore, flow cytometry analysis showed that apoptosis was increased in sh-THAP7-AS1-1 infected cells compared with sh-NC infected controls (Figure 2H). Collectively, these data indicate that THAP7-AS1 promotes the growth of liver cancer cells.

### Knockdown of THAP7-AS1 inhibits aerobic glycolysis in liver cancer cells

**FIGURE 3:**
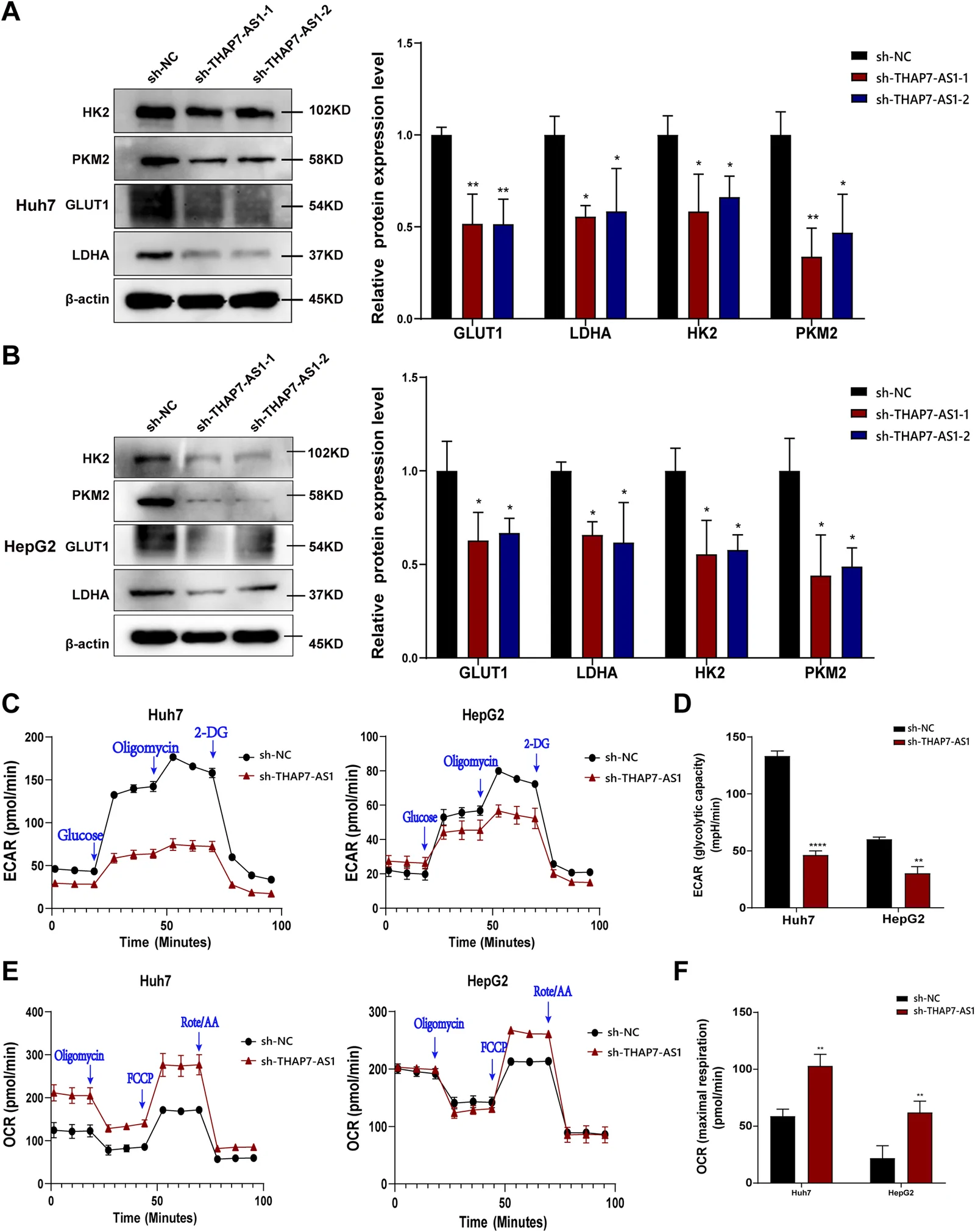
Knockdown of THAP7-AS1 inhibits aerobic glycolysis in liver cancer cells. **(A,B)** Western blot analysis showed that when THAP7-AS1 was inhibited, the expression levels of PFKFB2 and glycolytic genes, including GLUT1, LDHA, HK2 and PKM2, decreased (One-way ANOVA). **(C)** The Seahorse XF flux analyzer was used to detect ECAR in liver cancer cells (Huh7 and HepG2) from different groups (sh-THAP7-AS1 and sh-NC). Glucose, oligomycin and 2-DG were sequentially treated at different times. **(D)** Statistical analysis of the glycolytic activity shown in Panel **C** (Student’s t-test). **(E)** The Seahorse XF flux analyzer was used to detect OCR in liver cancer cells (Huh7 and HepG2) from different groups (sh-THAP7-AS1 and sh-NC). Oligomycin, FCCP and rotenone/antimycin A were sequentially treated at different times. **(F)** Statistical analysis of the maximum respiration shown in Panel **E** (Student’s t-test).*P < 0.05, **P < 0.01, ***P < 0.001, ****P < 0.0001.

The metabolic switch from oxidative phosphorylation to aerobic glycolysis, also termed the Warburg effect, facilitates tumor growth and suppresses apoptosis in cancer cells (Koppenol et al., 2011). To determine whether THAP7-AS1 promotes aerobic glycolysis in liver cancer cells, we examined the expression levels of several key glycolytic enzymes. Western blot analyses revealed that knockdown of THAP7-AS1 (sh-THAP7-AS1-1 and sh-THAP7-AS1-2) significantly reduced the expression of HK2, PKM2, GLUT1, and LDHA (Figures 3A,B). Subsequently, we selected sh-THAP7-AS1-1 for the subsequent functional assays. Furthermore, we employed XF technology to assess mitochondrial function by measuring the oxygen consumption rate (OCR) and glycolytic function by measuring the extracellular acidification rate (ECAR) in liver cancer cells (Plitzko and Loesgen, 2018). We next evaluated glycolytic flux by measuring ECAR using a metabolic flux analyzer. Knockdown of THAP7-AS1 significantly reduced ECAR in both Huh7 and HepG2 cells (Figures 3C,D). Mitochondrial respiration was assessed by measuring OCR to determine OXPHOS-dependent ATP production in Huh7 and HepG2 cells. Notably, OCR was enhanced in Huh7 and HepG2 cells following THAP7-AS1 knockdown (Figures 3E,F). Collectively, these results indicate that THAP7-AS1 promotes aerobic glycolysis while suppressing oxidative phosphorylation in liver cancer cells.

### Knocking down THAP7-AS1 inhibits tumor growth *in vivo*

**FIGURE 4:**
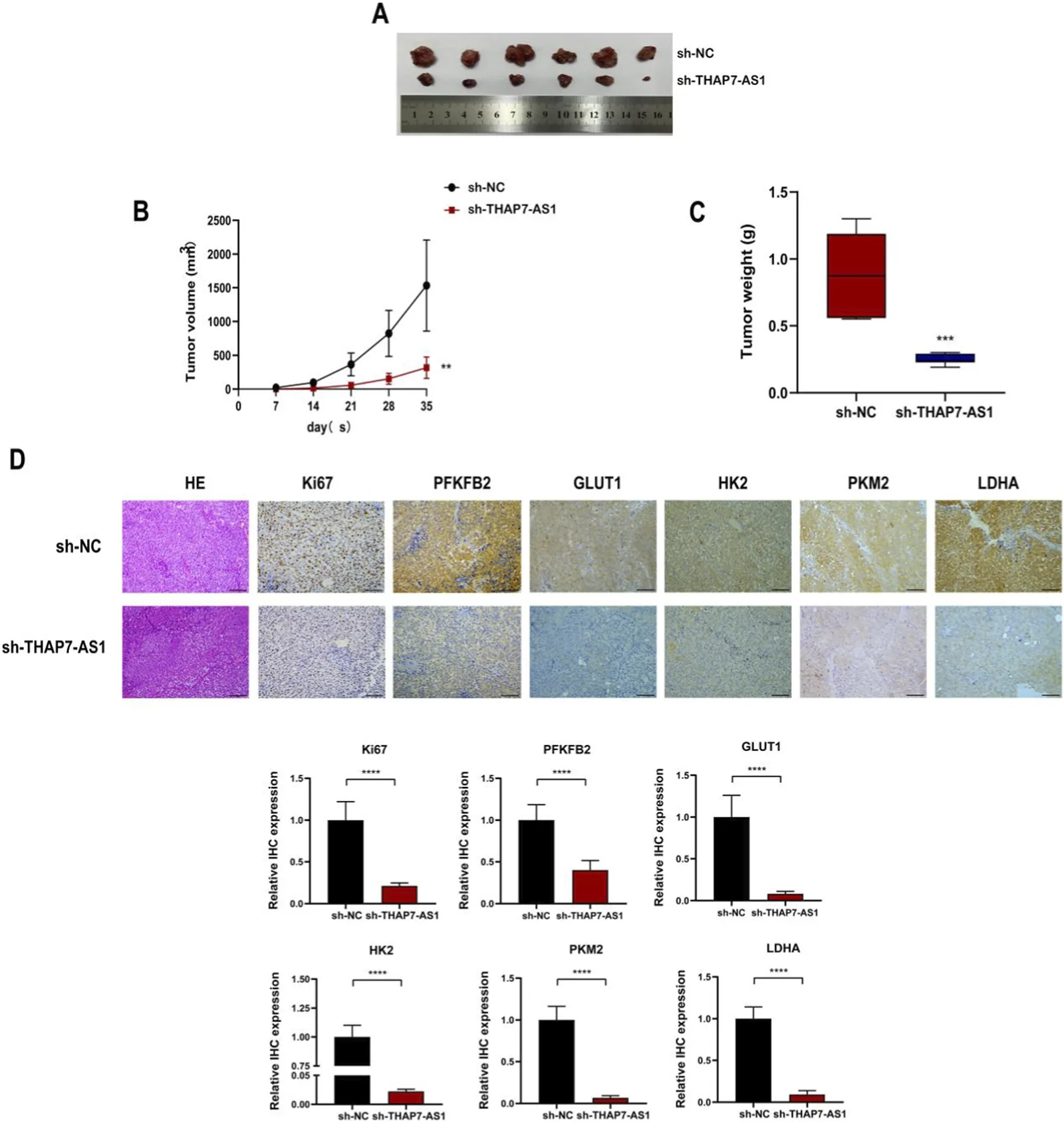
Knocking down THAP7-AS1 inhibits tumor growth *in vivo*. **(A)** Huh7-shNC or Huh7-shTHAP7-AS1 cells were inoculated subcutaneously on the right side of nude mice for tumor formation experiments. Seven weeks after transplantation, the tumor growth of nude mice was observed. **(B)** The tumor growth curve showed that compared with the sh-NC group, the sh-THAP7-AS1 group inhibited tumor growth (Student’s t-test). **(C)** Tumor weight (Student’s t-test). **(D)** The expression of Ki67, PFKFB2, GLUT1, HK2, PKM2 and LDHA was detected *in vivo* by immunohistochemical methods. Scale bar, 100 μm (Student’s t-test).*P < 0.05, **P < 0.01, ***P < 0.001, ****P < 0.0001.

To determine whether THAP7-AS1 promotes tumor growth *in vivo* via aerobic glycolysis, we performed a subcutaneous xenograft assay in BALB/c nude mice. Huh7 cells stably transfected with sh-THAP7-AS1 or sh-NC were injected into nude mice. Consistent with the *in vitro* findings, tumors in the sh-THAP7-AS1 group grew more slowly, with smaller volumes and lower weights compared with the sh-NC group (Figures 4A–C). Immunohistochemical (IHC) staining further revealed that the expression levels of Ki-67, PFKFB2, GLUT1, HK2, PKM2, and LDHA were decreased in the sh-THAP7-AS1group relative to the sh-NC group (Figure 4D) (100 μm). Collectively, these results demonstrate that THAP7-AS1 promotes *in vivo* tumor growth by enhancing aerobic glycolysis.

### THAP7-AS1 directly targeted miR-4286 in liver cancer cells

**FIGURE 5:**
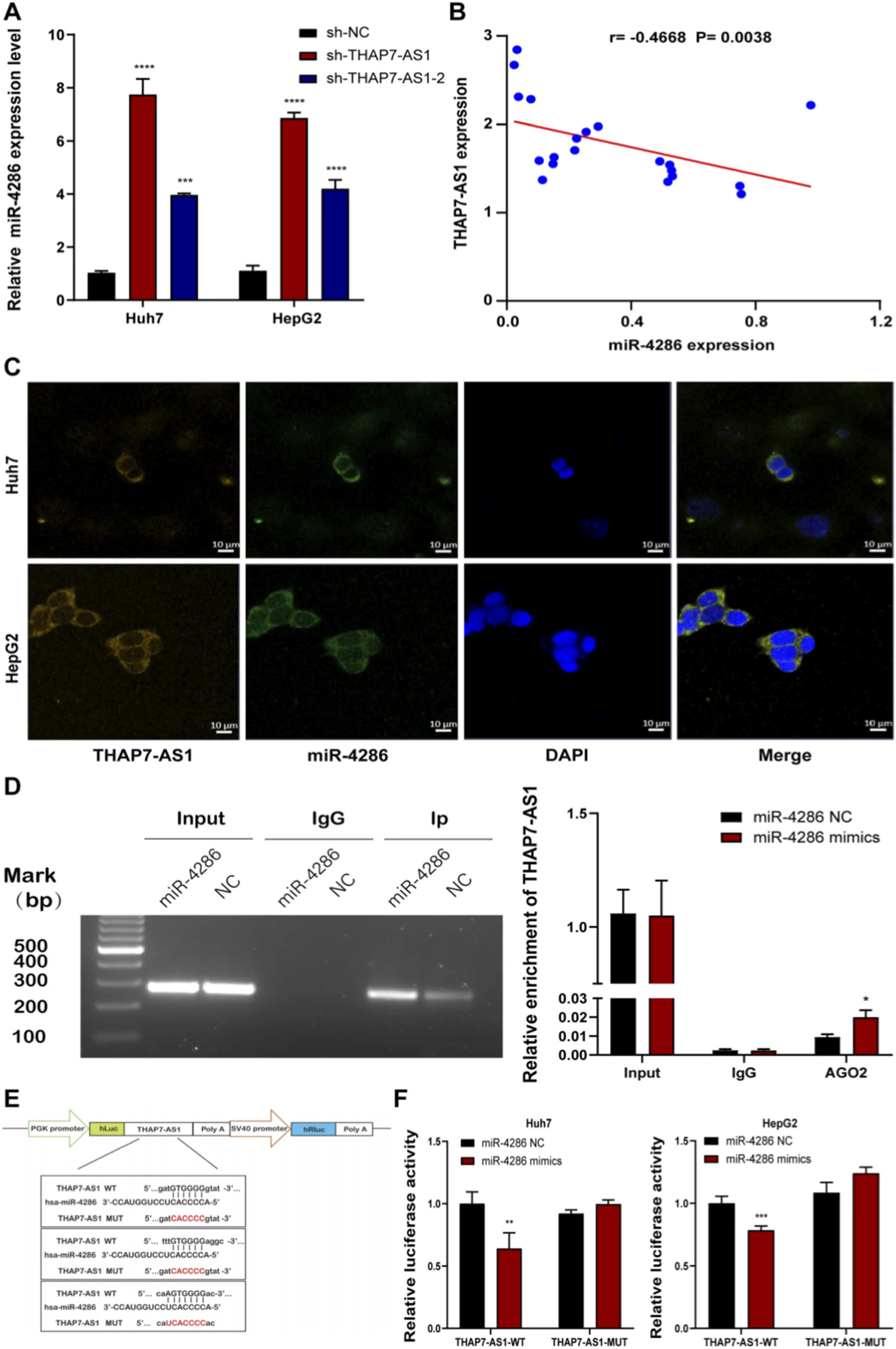
THAP7-AS1 directly targeted miR-4286 in liver cancer cells. **(A)** The RT-qPCR results showed that the expression of miR-4286 was upregulated in Huh7 and HepG2 cells transfected with sh-NC and sh-THAP7-AS1 (One-way ANOVA). **(B)** The expression levels of THAP7-AS1 and miR-4286 in 20 liver cancer tissues were detected by RT-qPCR. **(C)** The localization of THAP7-AS1 and miR-4286 in liver cancer cells was analyzed using RNA-FISH. The cell nucleus was stained with DAPI, with a scale of 10 μm. **(D)** RIP was used to determine whether miR-4286 binds to THAP7-AS1. The agarose gel electrophoresis figure and RT-qPCR results of the PCR product of THAP7-AS1. **(E)** Schematic diagram of the predicted binding sites between wild-type and mutant THAP7-AS1 and miR-4286. **(F)** Wild-type THAP7-AS1 3′-UTR (THAP7-AS1-WT) and mutant THAP7-AS1 3′-UTR (THAP7-AS1-MUT) were co-transfected with miR-4286 mimics or miR-4286 NC into Huh7 and HepG2 cells to detect luciferase activity (Student’s t-test).*P < 0.05, **P < 0.01, ***P < 0.001, ****P < 0.0001.

Existing studies suggest that lncRNAs can act as ceRNAs of miRNAs, by binding to the response elements and preventing miRNAs from inhibiting mRNAs (Chan and Tay, 2018). To verify whether THAP7-AS1 directly targets miR-4286 in liver cancer cells, we examined miR-4286 expression following THAP7-AS1 knockdown by RT-qPCR. The results showed that miR-4286 expression was significantly upregulated upon THAP7-AS1 silencing in liver cancer cells (Figure 5A). Further examination of miR-4286 expression levels in 20 pairs of liver cancer tissues and adjacent non-tumor tissues revealed a moderately negative correlation with THAP7-AS1 expression, and this association was statistically significant (r = −0.4668, *P* = 0.038; Figure 5B). RNA fluorescence *in situ* hybridization (FISH) revealed that THAP7-AS1 and miR-4286 were co-localized in the cytoplasm of liver cancer cells (Figure 5C). Notably, Ago2, an essential component of the RNA-induced silencing complex (RISC), was also evaluated to further explore the underlying mechanism (Bose et al., 2017). MiRNAs usually bind to their targets and inhibit translation and degradation of mRNAs in an Ago2-dependent manner (Lin et al., 2024). To further validate the interaction between THAP7-AS1 and miR-4286, RNA immunoprecipitation (RIP) assays were performed in 293T cells co-transfected with miR-4286 mimics or negative control (NC) and THAP7-AS1 wild-type (wt) or mutant (mut) constructs. Using anti-Ago2 antibody or control IgG for pulldown, we found that THAP7-AS1 enrichment by Ago2 was markedly increased in cells transfected with miR-4286 mimics compared with controls (Figure 5D). Bioinformatics analysis revealed multiple putative binding sites between THAP7-AS1 and miR-4286 (Figure 5E). Based on these predicted sites, we generated wild-type (pmirGLO-THAP7-AS1-wt) and mutant (pmirGLO-THAP7-AS1-mut) luciferase reporters, which were co-transfected into Huh7 and HepG2 cells along with miR-4286 mimics or NC. Dual-luciferase reporter assays demonstrated that overexpression of miR-4286 significantly reduced the luciferase activity of the wild-type reporter, whereas the mutant reporter remained unaffected (Figure 5F). Collectively, these data confirm that THAP7-AS1 directly targets and negatively regulates miR-4286 in liver cancer cells.

### MiR-4286 directly targeted PFKFB2 in liver cancer cells

**FIGURE 6:**
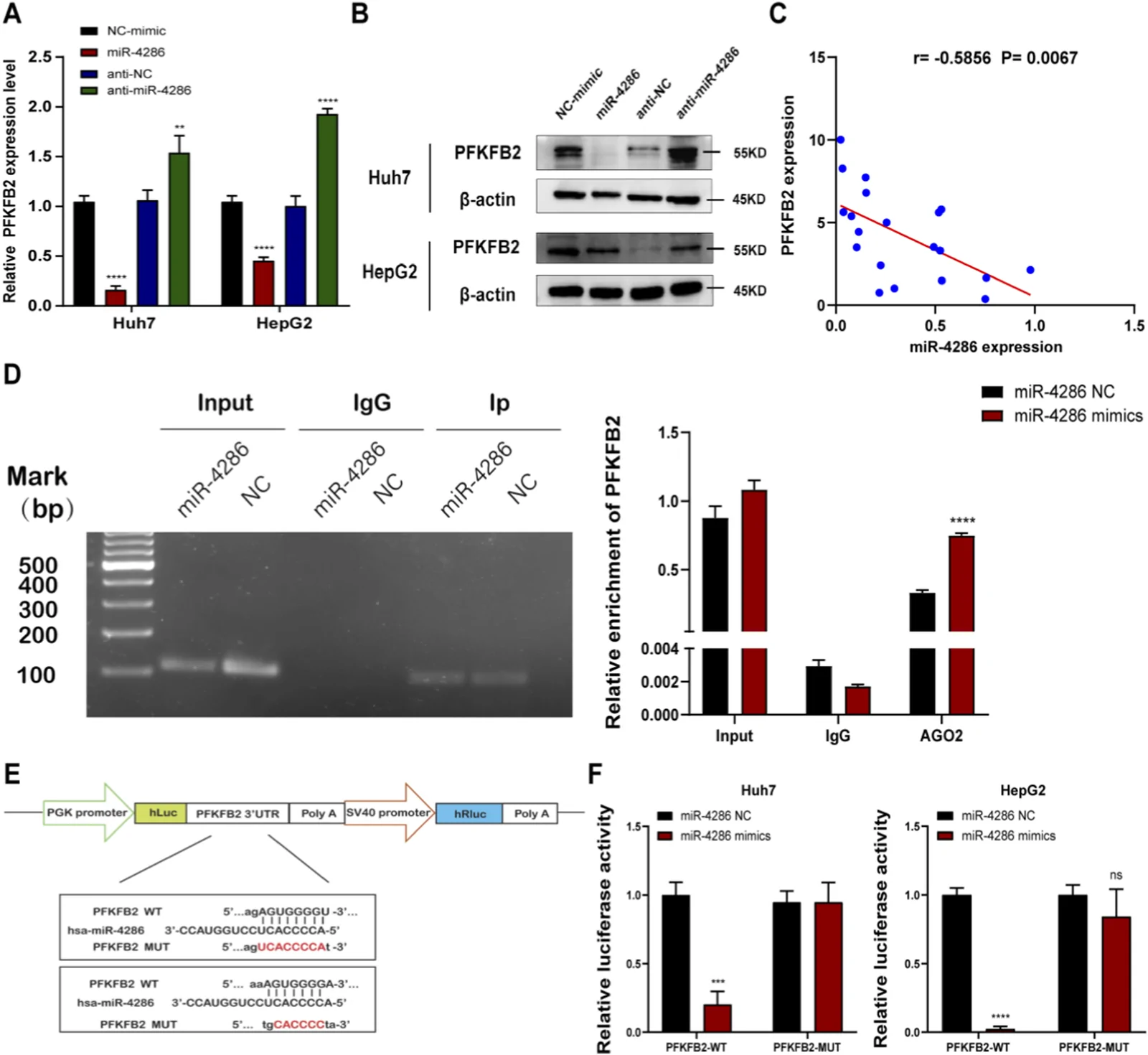
MiR-4286 directly targeted PFKFB2 in liver cancer cells. **(A)** RT-qPCR shows the expression of PFKFB2 mRNA in Huh7 and HepG2 cells transfected with miR-4286 mimics and miR-4286 NC (One-way ANOVA). **(B)** Western blot shows the expression of PFKFB2 in Huh7 and HepG2 cells transfected with miR-4286 mimics and miR-4286 NC. **(C)** The correlation between the expression of miR-4286 and PFKFB2 in liver cancer samples. **(D)** Determination of whether miR-4286 binds to PFKFB2 by RIP. The agarose gel electrophoresis figure and RT-qPCR results of PFKFB2’s qPCR products. **(E)** Schematic diagram of the predicted binding sites between wild-type and mutant PFKFB2 and miR-4286. **(F)** Co-transfection of wild-type PFKFB2 3′-UTR (PFKFB2-WT) and mutant PFKFB2 3′-UTR (PFKFB2-MUT) with miR-4286 mimics or miR-4286 NC in Huh7 and HepG2 cells to detect luciferase activity (Student’s t-test).*P < 0.05, **P < 0.01, ***P < 0.001, ****P < 0.0001.

We next investigated whether miR-4286 directly targets PFKFB2. To this end, Huh7 and HepG2 cells were transfected with miR-4286 mimics or inhibitors. Compared with controls, miR-4286 mimics significantly reduced both mRNA and protein levels of PFKFB2, whereas miR-4286 inhibitors markedly increased PFKFB2 expression at both the mRNA and protein levels (Figures 6A,B). Consistent with these findings, the expression of PFKFB2 in 20 pairs of clinical tissues exhibited a moderate negative correlation with miR-4286 levels, and this association was statistically significant (r = −0.5856, *P =* 0.0067; Figure 6C). RNA immunoprecipitation (RIP) assays further demonstrated that PFKFB2 enrichment by Ago2 was enhanced upon miR-4286 overexpression (Figure 6D). Bioinformatics analysis predicted putative miR-4286 binding sites within PFKFB2, and mutant reporters were generated accordingly (Figure 6E). Dual-luciferase reporter assays confirmed that miR-4286 directly targets PFKFB2: miR-4286 mimics significantly decreased the luciferase activity of the wild-type PFKFB2 reporter, whereas the mutant reporter remained unaffected in both Huh7 and HepG2 cells (Figure 6F). Collectively, these results establish PFKFB2 as a direct downstream target of miR-4286 and show that its expression is negatively regulated by miR-4286.

### Knockout of PFKFB2 inhibits the proliferation and glucose metabolism of liver cancer cells *in vitro*

**FIGURE 7:**
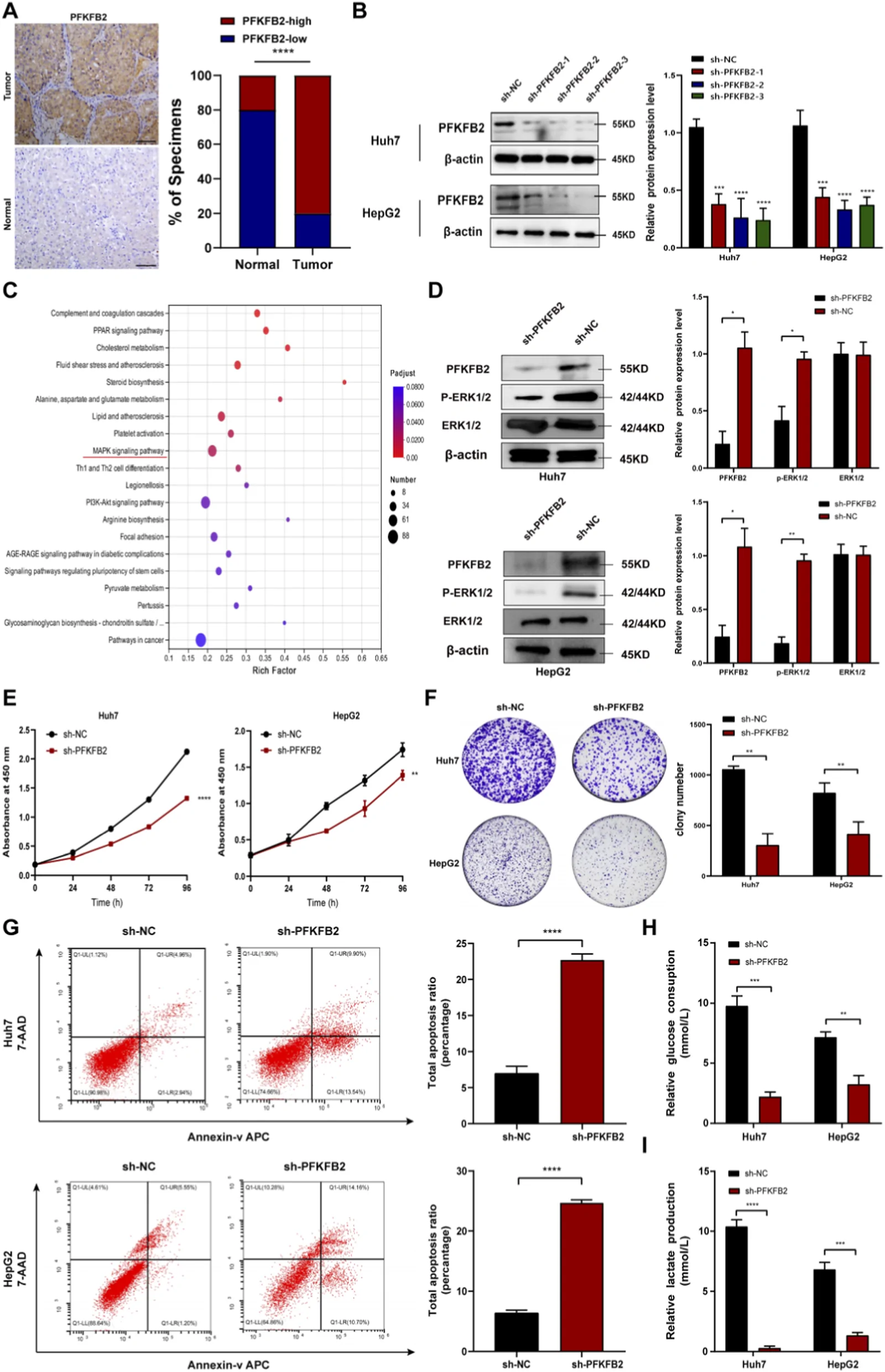
Knockout of PFKFB2 inhibits the proliferation and glucose metabolism of liver cancer cells *in vitro*. **(A)** Immunohistochemical method was used to detect the expression of PFKFB2 in 30 cases of liver cancer and adjacent tissues (Student’s t-test). **(B)** Western blot was employed to examine the expression of PFKFB2 in liver cancer cells after gene knockdown (One-way ANOVA). **(C)** KEGG enrichment analysis of high and low expression of PFKFB2 gene in Huh7 cells. **(D)** Protein blotting analysis was used to detect the changes in MAPK pathway-related proteins. **(E)** The CCK8 method shows that PFKFB2 gene knockdown inhibits the proliferation of liver cancer cells (Student’s t-test). **(F)** Colony formation assay was conducted to examine the effect of PFKFB2 knockdown on colony formation ability of Huh7 and HepG2 cells (Student’s t-test). **(G)** FCM of apoptosis levels (Student’s t-test). **(H)** Detection of glucose consumption levels in Huh7 and HepG2 cells after PFKFB2 knockdown (Student’s t-test). **(I)** Lactate production was determined in the Huh7 and HepG2 cells stable knockdown PFKFB2 (Student’s t-test).*P < 0.05, **P < 0.01, ***P < 0.001, ****P < 0.0001.

PFKFB2 is a key enzyme that regulates the synthesis and degradation of fructose-2,6-bisphosphate (F-2,6-BP) during glycolysis. Previous studies have linked PFKFB activity to glycolytic flux, cell proliferation, cell survival, and tumor growth (Pan et al., 2020; Qu et al., 2020). PFKFB2 has also been demonstrated to be abnormally expressed in various types of tumors, including pancreatic cancer (Ozcan et al., 2020), gastric cancer (L et al., 2019), liver cancer (Ji et al., 2014), etc. To explore the biological function of PFKFB2 in liver cancer cells, we first confirmed by immunohistochemistry (IHC) that PFKFB2 expression was upregulated in liver cancer tissues compared with corresponding adjacent non-tumor tissues in 30 paired clinical samples (Figure 7A). We then infected Huh7 and HepG2 cells with sh-NC or sh-PFKFB2 and verified the knockdown efficiency (Figure 7B). Kyoto Encyclopedia of Genes and Genomes (KEGG) pathway analysis revealed that differentially expressed genes (DEGs) following sh-PFKFB2 group in Huh7 cells were predominantly enriched in the MAPK signaling pathway (Figure 7C). Notably, previous studies have reported that lncRNAs can promote ERK1/2 phosphorylation, activate the MAPK pathway, and enhance liver cancer cell proliferation and tumor growth-findings that are consistent with our results (Chen et al., 2018). Consistent with the Western blotting results, knockdown of PFKFB2 significantly suppressed the expression of MAPK/ERK1/2 signaling pathway-related proteins in liver cancer cells (Figure 7D), suggesting that PFKFB2 may exert its function through this pathway. We further assessed the functional consequences of PFKFB2 knockdown using CCK-8 and colony formation assays, which revealed that PFKFB2 silencing weakened the proliferation of Huh7 and HepG2 cells (Figures 7E,F). Flow cytometry analysis further demonstrated that PFKFB2 knockdown significantly induced apoptosis in liver cancer cells compared with the control group (Figure 7G). Moreover, glucose consumption and lactate production were markedly reduced following PFKFB2 knockdown (Figures 7H,I). Collectively, these results indicate that PFKFB2 knockdown inhibits the proliferation and glucose metabolism of liver cancer cells *in vitro*.

### THAP7-AS1 regulates miR-4286 through PFKFB2, thereby enhancing aerobic glycolysis and promoting the growth of liver cancer cells

**FIGURE 8:**
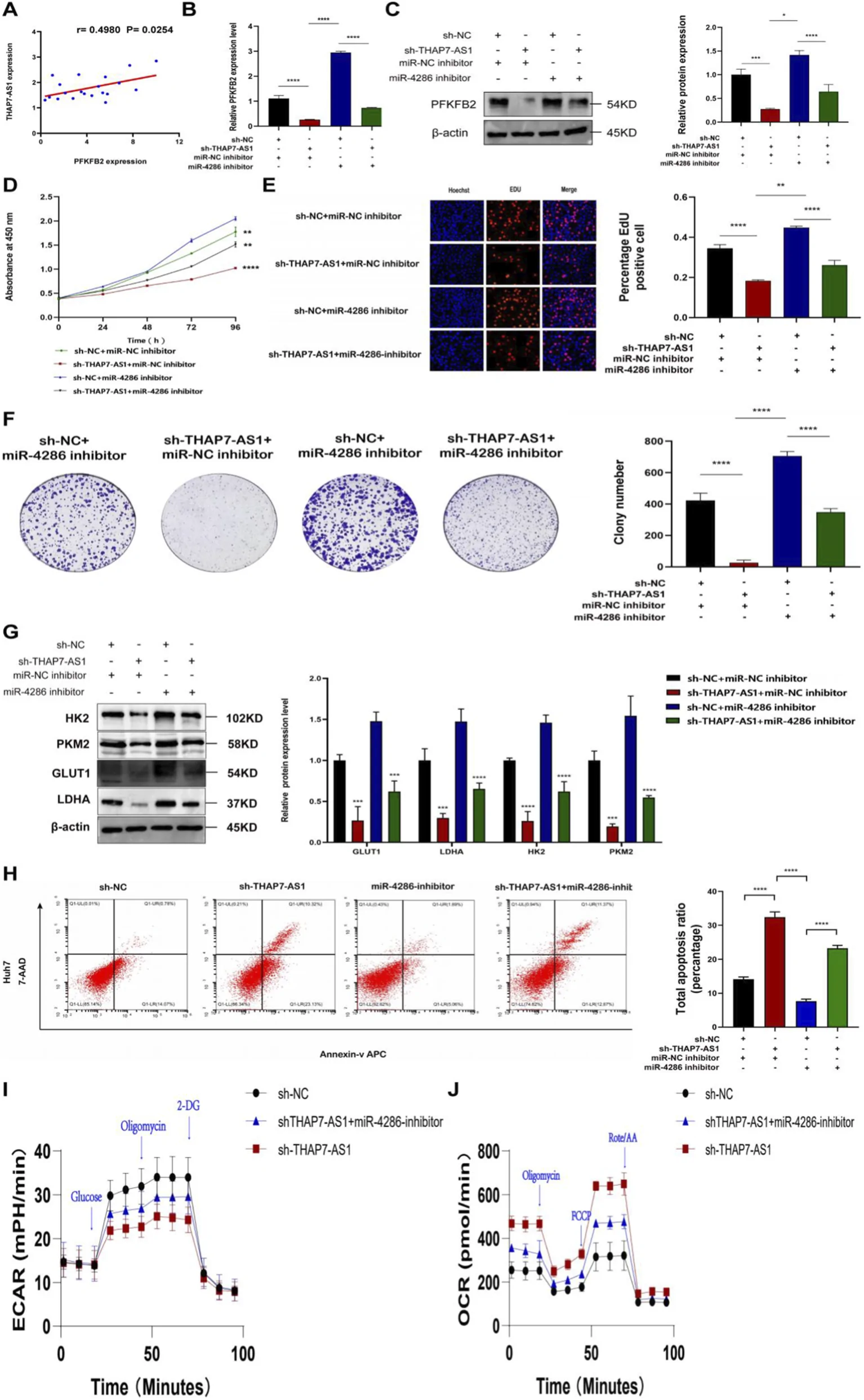
THAP7-AS1 regulates miR-4286 through PFKFB2, thereby enhancing aerobic glycolysis and promoting the growth of liver cancer cells. **(A)** Correlation between the expression of THAP7-AS1 and PFKFB2 mRNA in 20 liver cancer tissues. **(B,C)** RT-qPCR and Western blot showed the expression of PFKFB2 in Huh7 cells transfected with miR-4286 inhibitor, sh-THAP7-AS1, or sh-NC (One-way ANOVA). **(D–F)** CCK-8, EDU, and colony formation experiments demonstrated that miR-4286 gene knockdown could partially reverse the inhibitory effect of THAP7-AS1 gene knockdown on the proliferation of liver cancer cells (One-way ANOVA). **(G)** Western blot experiment showed that miR-4286 gene knockdown partially reversed the inhibition of THAP7-AS1 expression in liver cancer cells for PFKFB2 and glycolytic enzymes HK2, PKM2, GLUT1, and LDHA (One-way ANOVA). **(H)** FCM of apoptosis levels. **(I)** The ECAR in different groups (miR-4286 inhibitor, sh-THAP7-AS1, or negative control) was detected using the Seahorse XF cell flux analyzer. Different times were treated with glucose, oligomycin, and 2-DG successively (One-way ANOVA). **(J)** The OCR in different groups (miR-4286 inhibitor, sh-THAP7-AS1, or negative control) was detected using the Seahorse XF cell flux analyzer. Different times were treated with oligomycin, FCCP, and rotenone/antimycin A successively.*P < 0.05, **P < 0.01, ***P < 0.001, ****P < 0.0001.

**FIGURE 9:**
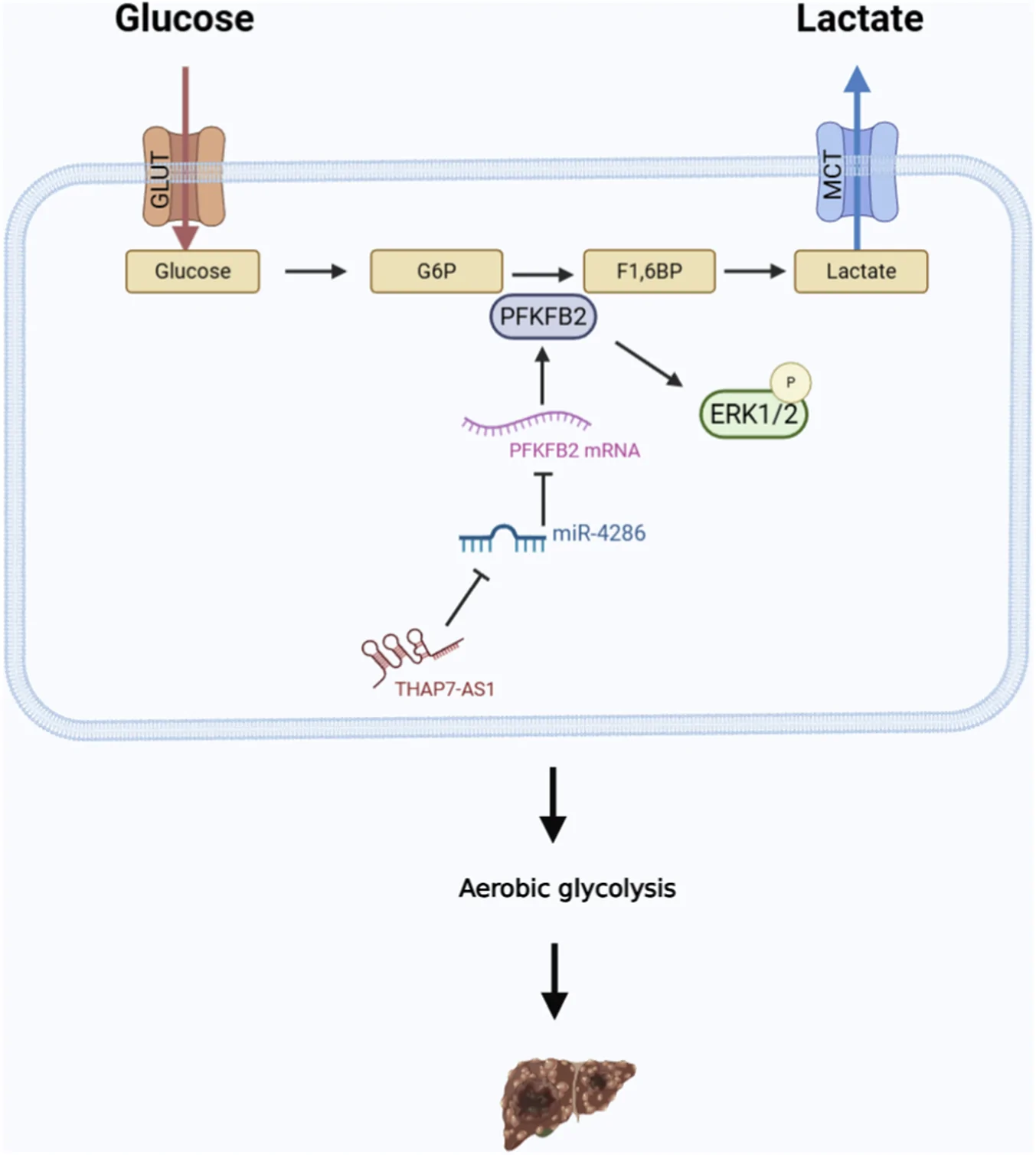
Proposed model for the functional landscape of the THAP7-AS1/miR-4286/PFKFB2 in liver cancer cell pathway as a key mediator of these effects. These findings suggest a pro-tumorigenic role for PFKFB2 via the MAPK signaling pathway in liver cancer. However, this study has certain limitations. In tissue samples, the correlations among THAP7-AS1, miR-4286, and PFKFB2 expression were only moderate, suggesting that additional molecules may be involved in regulating PFKFB2 expression in clinical specimens. Further experiments are warranted to explore these potential regulatory factors.

To investigate the relationship between the THAP7-AS1/miR-4286/PFKFB2 axis and the aberrant proliferation and glucose metabolism of liver cancer cells, we analyzed THAP7-AS1 and PFKFB2 mRNA expression levels in 20 liver cancer tissues. The results demonstrated a moderate positive correlation between THAP7-AS1 expression and PFKFB2 mRNA expression (r = 0.4980, *P* = 0.0254; Figure 8A). RT-qPCR and Western blotting assays demonstrated that PFKFB2 expression was significantly reduced upon THAP7-AS1 knockdown, whereas it was restored in cells co-transfected with sh-THAP7-AS1-1 and miR-4286 inhibitor (Figures 8B,C). Functional assays showed that miR-4286 knockdown attenuated the inhibitory effects of THAP7-AS1 silencing on liver cancer cell growth, as assessed by CCK-8 (Figure 8D), EdU incorporation (Figure 8E), and colony formation assays (Figure 8F). Furthermore, the expression levels of glycolytic enzymes downstream of THAP7-AS1, including HK2, PKM2, GLUT1, and LDHA, were consistently decreased upon THAP7-AS1 knockdown, whereas their expression was restored in cells co-transfected with sh-THAP7-AS1-1 and miR-4286 inhibitor (Figure 8G). Flow cytometry analysis further confirmed that miR-4286 knockdown attenuated the growth-inhibitory effect of THAP7-AS1 silencing (Figure 8H). Moreover, co-transfection with sh-THAP7-AS1-1 and miR-4286 inhibitor enhanced the Warburg effect in liver cancer cells compared with sh-THAP7-AS1-1 alone (Figures 8I,J). Collectively, these results demonstrate that THAP7-AS1 functions as a ceRNA that promotes tumor cell growth and the Warburg effect by sponging miR-4286 and subsequently upregulating PFKFB2 expression (Figure 9).

## Discussion

Clinical evidence has demonstrated that high expression of lncRNAs is associated with increased recurrence rates and shorter overall survival in liver cancer patients (Gao et al., 2013; Lin et al., 2018). This finding offers a novel perspective for the exploration of molecular mechanisms underlying liver cancer pathogenesis. Serum circulating lncRNAs represent minimally invasive liquid biopsy biomarkers that can replace invasive tissue biopsies. Their accessibility through repeated serum sampling enables longitudinal monitoring of disease progression. Moreover, circulating lncRNAs can capture early molecular aberrations in liver cancer, facilitating early diagnosis and real-time assessment of treatment response (You et al., 2023). This study confirms that elevated THAP7-AS1 levels in the serum of liver cancer patients hold promise as a non-invasive serum biomarker for clinical detection of liver cancer. Given that extracellular lncRNAs detected in serum are largely derived from exosomes released by tumor cells or from cell-free nucleic acids, serum molecular abnormalities may indirectly reflect transcriptional alterations within liver cancer lesions (Qi et al., 2016). Therefore, following the identification of differentially expressed THAP7-AS1 in serum, we examined its expression level in tumor tissues to determine whether the aberrantly expressed molecules in serum truly originated from liver cancer lesions. Our findings confirmed that THAP7-AS1 expression was significantly upregulated in liver cancer tissues and correlated with poor prognosis in liver cancer patients. Additionally, THAP7-AS1 promoted cell proliferation, inhibited apoptosis, and enhanced tumor growth *in vitro*. Collectively, these findings indicate that THAP7-AS1 may function as a pro-umorigenic factor in liver cancer.

It is now well established that increased glucose consumption and lactate production by tumor cells provide the carbon sources required for the growth of proliferating cancer cells (Bhattacharya et al., 2016; Kobayashi et al., 2019). Aerobic glycolysis is thus fundamental to tumor initiation and progression, and investigating its regulatory mechanisms holds significant translational potential. Targeting aerobic glycolysis may represent a promising therapeutic strategy for liver cancer. Our findings demonstrate that THAP7-AS1 knockdown markedly reduces aerobic glycolysis in liver cancer cells, underscoring the importance of elucidating the metabolic functions of lncRNAs. Previous studies have reported that certain lncRNAs in liver cancer act as miRNA sponges, thereby modulating the ceRNA network that regulates downstream protein-coding gene expression. Collectively, these observationsOur findings demonstrate that PFKFB2 expression is markedly elevated in liver cancer tissues. Knockdown of PFKFB2 significantly suppresses liver cancer cell proliferation and reduces both glucose consumption and lactate production. Mechanistically, we identified the MAPK/ERK1/2.

In summary, this study identifies THAP7-AS1 as a highly expressed oncogene in liver cancer. Its elevated levels in the serum of liver cancer patients hold promise as a non-invasive biomarker for clinical detection. Mechanistically, we demonstrate that THAP7-AS1 competitively binds to miR-4286, thereby upregulating PFKFB2 expression and modulating glucose metabolic reprogramming via the MAPK/ERK1/2 signaling pathway. Collectively, these findings suggest that THAP7-AS1 may serve as a potential therapeutic target for liver cancer.

## Data Availability

The raw data supporting the conclusions of this article will be made available by the authors, without undue reservation.

## References

[B1] Balihodzic A. Barth D. A. Prinz F. Pichler M. (2021). Involvement of long non-coding RNAs in glucose metabolism in cancer. Cancers (Basel) 13 (5), 977. 10.3390/cancers13050977 33652661 PMC7956509

[B2] Bhattacharya B. Mohd O. M. Soong R. (2016). The warburg effect and drug resistance. Br. J. Pharmacol. 173 (6), 970–979. 10.1111/bph.13422 26750865 PMC4793921

[B3] Bose M. Barman B. Goswami A. Bhattacharyya S. N. (2017). Spatiotemporal uncoupling of MicroRNA-Mediated translational repression and target RNA degradation controls MicroRNP recycling in mammalian cells. Mol. Cell Biol. 37 (4), e00464–16. 10.1128/MCB.00464-16 27895152 PMC5288578

[B4] Bray F. Ferlay J. Soerjomataram I. Siegel R. L. Torre L. A. Jemal A. (2018). Global cancer statistics 2018: GLOBOCAN estimates of incidence and mortality worldwide for 36 cancers in 185 countries. CA Cancer J. Clin. 68 (6), 394–424. 10.3322/caac.21492 30207593

[B5] Chan J. J. Tay Y. (2018). Noncoding RNA:RNA regulatory networks in cancer. Int. J. Mol. Sci. 19 (5), 1310. 10.3390/ijms19051310 29702599 PMC5983611

[B6] Chen T. Huang H. Zhou Y. Geng L. Shen T. Yin S. (2018). HJURP promotes hepatocellular carcinoma proliferation by destabilizing p21 via the MAPK/ERK1/2 and AKT/GSK3β signaling pathways. J. Exp. Clin. Cancer Res. 37 (1), 193. 10.1186/s13046-018-0866-4 30111352 PMC6094877

[B7] DeBerardinis R. J. Lum J. J. Hatzivassiliou G. Thompson C. B. (2008). The biology of cancer: metabolic reprogramming fuels cell growth and proliferation. Cell Metab. 7 (1), 11–20. 10.1016/j.cmet.2007.10.002 18177721

[B8] Derrien T. Johnson R. Bussotti G. Tanzer A. Djebali S. Tilgner H. (2012). The GENCODE v7 catalog of human long noncoding RNAs: analysis of their gene structure, evolution, and expression. Genome Res. 22 (9), 1775–1789. 10.1101/gr.132159.111 22955988 PMC3431493

[B9] Gao X. Huang M. Liu L. He Y. Yu Q. Zhao H. (2013). Insertion/deletion polymorphisms in the promoter region of BRM contribute to risk of hepatocellular carcinoma in Chinese populations. PLoS One 8 (1), e55169. 10.1371/journal.pone.0055169 23359823 PMC3554679

[B10] Hanahan D. Weinberg R. A. (2011). Hallmarks of cancer: the next generation. Cell 144 (5), 646–674. 10.1016/j.cell.2011.02.013 21376230

[B11] Hsu P. P. Sabatini D. M. (2008). Cancer cell metabolism: warburg and beyond. Cell 134 (5), 703–707. 10.1016/j.cell.2008.08.021 18775299

[B12] Hua Q. Jin M. Mi B. Xu F. Li T. Zhao L. (2019). LINC01123, a c-Myc-activated long non-coding RNA, promotes proliferation and aerobic glycolysis of non-small cell lung cancer through miR-199a-5p/c-Myc axis. J. Hematol. Oncol. 12 (1), 91. 10.1186/s13045-019-0773-y 31488218 PMC6728969

[B13] Huang Q. Liang Z. Huang Q. Li X. Xia J. Huang L. (2024). Involvement of lncRNAs in the regulation of aerobic glycolysis in hepatocellular carcinoma: main functions, regulatory mechanisms and potential therapeutic implications. Oncol. Rep. 51 (6), 84. 10.3892/or.2024.8743 38666534 PMC11082637

[B14] Ji D. Lu Z. T. Li Y. Q. Liang Z. Y. Zhang P. F. Li C. (2014). MACC1 expression correlates with PFKFB2 and survival in hepatocellular carcinoma. Asian Pac J. Cancer Prev. 15 (2), 999–1003. 10.7314/apjcp.2014.15.2.999 24568531

[B15] Kobayashi Y. Banno K. Kunitomi H. Takahashi T. Takeda T. Nakamura K. (2019). Warburg effect in gynecologic cancers. J. Obstet. Gynaecol. Res. 45 (3), 542–548. 10.1111/jog.13867 30511455

[B16] Koppenol W. H. Bounds P. L. Dang C. V. (2011). Otto Warburg's contributions to current concepts of cancer metabolism. Nat. Rev. Cancer 11 (5), 325–337. 10.1038/nrc3038 21508971

[B17] Liu H. Chen K. Wang L. Zeng X. Huang Z. Li M. (2019). miR-613 inhibits warburg effect in gastric cancer by targeting PFKFB2. Biochem. Biophys. Res. Commun. 515 (1), 37–43. 10.1016/j.bbrc.2019.05.001 31122697

[B18] Li Z. Liu Y. Hou Y. Chen C. Hao H. Liu Y. (2022). Construction and function analysis of the LncRNA-miRNA-mRNA competing endogenous RNA network in autoimmune hepatitis. BMC Med. Genomics 15 (1), 270. 10.1186/s12920-022-01416-4 36566205 PMC9790135

[B19] Li Q. Bai J. Zheng S. Liao J. Yang Z. Cai W. (2026). THAP7-AS1 orchestrates IGF2BP3-m(6)A-dependent CCN2 mRNA stabilization to promote lymphatic metastasis in bladder cancer. Am. J. Cancer Res. 16 (3), 1215–1230. 10.62347/WGQA3188 42004053 PMC13090477

[B20] Lin Y. H. Wu M. H. Yeh C. T. Lin K. H. (2018). Long non-coding RNAs as mediators of tumor microenvironment and liver cancer cell communication. Int. J. Mol. Sci. 19 (12), 3742. 10.3390/ijms19123742 30477236 PMC6321423

[B21] Lin M. C. Kuo W. H. Chen S. Y. Hsu J. Y. Lu L. Y. Wang C. C. (2024). Ago2/CAV1 interaction potentiates metastasis via controlling Ago2 localization and miRNA action. EMBO Rep. 25 (5), 2441–2478. 10.1038/s44319-024-00132-7 38649663 PMC11094075

[B22] Liu H. T. Zou Y. X. Zhu W. J. Liu S. Zhang G. h. Ma R. R. (2022). lncRNA THAP7-AS1, transcriptionally activated by SP1 and post-transcriptionally stabilized by METTL3-mediated m6A modification, exerts oncogenic properties by improving CUL4B entry into the nucleus. Cell Death Differ. 29 (3), 627–641. 10.1038/s41418-021-00879-9 34608273 PMC8901790

[B23] Marrero J. A. Kulik L. M. Sirlin C. B. Zhu A. X. Finn R. S. Abecassis M. M. (2018). Diagnosis, staging, and management of hepatocellular carcinoma: 2018 practice guidance by the American association for the study of liver diseases. Hepatology 68 (2), 723–750. 10.1002/hep.29913 29624699

[B25] Ozcan S. C. Sarioglu A. Altunok T. H. Akkoc A. Guzel S. Guler S. (2020). PFKFB2 regulates glycolysis and proliferation in pancreatic cancer cells. Mol. Cell Biochem. 470 (1-2), 115–129. 10.1007/s11010-020-03751-5 32415418

[B26] Pan X. Li H. Tan J. Weng X. Zhou L. Weng Y. (2020). miR-1297 suppresses osteosarcoma proliferation and aerobic glycolysis by regulating PFKFB2. Onco Targets Ther. 13, 11265–11275. 10.2147/OTT.S274744 33173315 PMC7648564

[B27] Pardini B. Sabo A. A. Birolo G. Calin G. A. (2019). Noncoding RNAs in extracellular fluids as cancer biomarkers: the new frontier of liquid biopsies. Cancers (Basel) 11 (8), 1170. 10.3390/cancers11081170 31416190 PMC6721601

[B28] Plitzko B. Loesgen S. (2018). Measurement of oxygen consumption rate (OCR) and extracellular acidification rate (ECAR) in culture cells for assessment of the energy metabolism. Bio Protoc. 8 (10), e2850. 10.21769/BioProtoc.2850 34285967 PMC8275291

[B29] Qi P. Zhou X. Y. Du X. (2016). Circulating long non-coding RNAs in cancer: current status and future perspectives. Mol. Cancer 15 (1), 39. 10.1186/s12943-016-0524-4 27189224 PMC4869386

[B30] Qu J. Yang J. Chen M. Wei R. Tian J. (2020). CircFLNA acts as a sponge of miR-646 to facilitate the proliferation, metastasis, glycolysis, and apoptosis inhibition of gastric cancer by targeting PFKFB2. Cancer Manag. Res. 12, 8093–8103. 10.2147/CMAR.S264674 32982406 PMC7490063

[B31] Rao A. Rajkumar T. Mani S. (2017). Perspectives of long non-coding RNAs in cancer. Mol. Biol. Rep. 44 (2), 203–218. 10.1007/s11033-017-4103-6 28391434

[B32] Shen Y. Xu J. Pan X. Zhang Y. Weng Y. Zhou D. (2020). LncRNA KCNQ1OT1 sponges miR-34c-5p to promote osteosarcoma growth *via* ALDOA enhanced aerobic glycolysis. Cell Death Dis. 11 (4), 278. 10.1038/s41419-020-2485-1 32332718 PMC7181648

[B33] Statello L. Guo C. J. Chen L. L. Huarte M. (2021). Gene regulation by long non-coding RNAs and its biological functions. Nat. Rev. Mol. Cell Biol. 22 (2), 96–118. 10.1038/s41580-020-00315-9 33353982 PMC7754182

[B35] You J. Xia H. Huang Z. He R. Zhao X. Chen J. (2023). Research progress of circulating non-coding RNA in diagnosis and treatment of hepatocellular carcinoma. Front. Oncol. 13, 1204715. 10.3389/fonc.2023.1204715 37546394 PMC10400719

